# Identification and evaluation of suitable reference genes for gene expression studies in the whitefly *Bemisia tabaci* (Asia I) by reverse transcription quantitative realtime PCR

**DOI:** 10.1093/jis/14.1.63

**Published:** 2014-01-01

**Authors:** Carl Collins, Mitulkumar V. Patel, John Colvin, David Bailey, Susan Seal

**Affiliations:** Natural Resources Institute, University of Greenwich at Medway, Central Avenue, Chatham Maritime, Kent, ME4 4TB, United Kingdom

## Abstract

This study presents a reliable method for performing reverse transcription quantitative realtime PCR (RT-qPCR) to measure gene expression in the whitefly *Bemisia tabaci* (Asia I) (Gennadius) (Hemiptera: Aleyrodidae), utilising suitable reference genes for data normalisation. We identified orthologs of commonly used reference genes (actin (ACT), cyclophilin 1 (CYP1), elongation factor 1α (EF1A), glyceraldehyde 3-phosphate dehydrogenase (GAPDH), ribosomal protein L13a (RPL13A), and α-tubulin (TUB1A)), measured the levels of their transcripts by RT-qPCR during development and in response to thermal stress, and evaluated their suitability as endogenous controls using geNorm, BestKeeper, and NormFinder programs. Overall, TUB1A, RPL13A, and CYP1 were the most stable reference genes during *B. tabaci* development, and TUB1A, GAPDH, and RPL13A were the most stable reference genes in the context of thermal stress. An analysis of the effects of reference gene choice on the transcript profile of a developmentally-regulated gene encoding vitellogenin demonstrated the importance of selecting the correct endogenous controls for RT-qPCR studies. We propose the use of TUB1A, RPL13A, and CYP1 as endogenous controls for transcript profiling studies of *B. tabaci* development, whereas the combination of TUB1A, GAPDH, and RPL13A should be employed for studies into thermal stress. The data pre- sented here will assist future transcript profiling studies in whiteflies.

## Introduction

The whitefly *Bemisia tabaci* (Gennadius) (Hemiptera: Aleyrodidae) species complex encompasses some of the most invasive and destructive pests of agricultural and ornamental crops throughout the world and is responsible for losses of billions of dollars annually ( Brown et al. 1995 ; Oliveira et al. 2001 ; Dalton et al. 2006 ; Seal et al. 2006 ). The whitefly represent a major threat to global food production primarily due to their direct damaging effects on host plants, their broad host range, and their ability to vector numerous plant viruses ( Bedford et al. 1994 ; Seal et al. 2006 ; Crowder et al. 2009 ). As a consequence of the growing economic importance of *B. tabaci* worldwide, genomic resources have been developed utilising next generation sequencing technologies for whitefly transcriptome assembly ( Leshkowitz et al. 2006 ; Wang et al. 2010 ; Seal et al. 2012 ; Xie et al. 2012 ). Despite the absence of a complete genome for *B. tabaci,* the available expressed sequence tag (EST) datasets have enabled the development of methods for the analysis of gene expression, such as microarray and reverse transcription quantitative realtime PCR (RT-qPCR), to help elucidate molecular mechanisms of insecticide resistance, thermotolerance, and parasitoid-host interactions ( Leshkowitz et al. 2006 ; Ghanim and Kontsedalov 2007 ; Mahadav et al. 2008 , 2009; Yu and Wan 2009 ; Wang et al. 2010 ; Seal et al. 2012 ; Xie et al. 2012 ). The anticipated growth in post-genomic studies in *B. tabaci* means that RT-qPCR-based methods will continue to offer a powerful means by which to identify differentially-expressed genes involved in fundamental biological processes, particularly now that high- throughput RT-qPCR methodologies are available ( Morrison et al. 2006 ).

RT-qPCR is the most sensitive, accurate, and reproducible quantitative method for the analysis of gene expression and is employed extensively for the verification of data derived from high-throughput microarray studies. For accurate and reliable gene expression analysis, it is critical that RT-qPCR data is normalised appropriately to control for variations in the efficiency of reverse transcription and PCR, and the purity and integrity of mRNA. The most common strategy for normalisation involves relating the mRNA abundance of the gene of interest to that of a single endogenous reference gene and is based on the assumption that the reference gene is expressed constituti- vely and is independent of cell or tissue type, or different biological or treatment conditions ( Pfaffl 2001 ). However, significant constraints to this approach exist that can undermine the accuracy of RT-qPCR data. For example, the mRNA levels of commonly-used reference genes such as actin (ACT) and glyceraldehyde 3-phosphate dehydrogenase (GAPDH) have been shown to vary significantly under particular developmental contexts and experimental conditions ( Radonić et al. 2004 ; De Jonge et al. 2007 ), and the use of only a single reference gene as internal control can lead to erroneous normalisation with transcript levels differing up to 20-fold ( Vandesompele et al. 2002 ). Therefore, for accurate normalisation it is vital that potential reference genes be validated for each species and experimental conditions employed to assure stability of expression in advance of relative quantification of target genes by RT-qPCR.

Software-based methods have been developed that allow a more rational approach to the selection and standardisation of endogenous controls for RT-qPCR studies. The three software programs geNorm ( Vandesompele et al. 2002 ), NormFinder (Anderson et al. 2004), and BestKeeper ( Pfaffl et al. 2004 ) use statistical algorithms to calculate stability measurements for a given set of reference genes that ranks each gene from the most stably expressed to the least stably expressed reference gene, and supports the use of multiple normalisation genes. No systematic survey comparing the suitability of different candidate reference genes has been conducted in *B. tabaci,* and of the transcript profiling studies that have been published to date, all but one have made use of a single, unvalidated normalisation gene ( Sinisterra et al. 2005 ; Alon et al. 2008 ; Karunker et al. 2008 ; Mahadav et al. 2008 , 2009; Mason et al. 2008 ; Yu and Wan 2009 ). The absence of fully-tested, reliable internal reference genes in *B. tabaci* makes the establishment of more precise methods for standardisation of RT-qPCR an urgent requirement in whitefly research.

Therefore, the aim of this study was to identify candidate reference genes in *B. tabaci* and to assess which of these genes are the most suitable for normalisation of RT-qPCR data during development, in different adult tissues, and in response to thermal stress. In addition, we identified and characterised by transcription profiling a developmentally-regulated gene that showed high homology to vitellogenin genes from other insects and served as a developmental marker to validate the transcription profiles observed for the reference genes during development. We also examined the effects of reference gene selection upon the calculated expression profile of the vitellogenin gene during development and in different adult tissues to demonstrate the importance of adopting the correct normalisation strategy for analysing gene expression. The data presented here will facilitate accurate and reproducible transcript profiling studies in *B. tabaci* during development and in response to thermal stress and will provide a useful resource for selection of reference genes in different tissues and experimental conditions in *B. tabaci* and other closely-related insects.

## Materials and Methods

### Insects and rearing conditions

The *B. tabaci* (Asia I genetic clade) population used in this study were reared on cotton plants, *Gossypium hirsutum* L. (Cv. Laxmi) (Malvales: Malvaceae), in insect-proof cages under controlled conditions of temperature (26 ± 1°C), light (14:10 L:D), and relative humidity (70 ± 10%). The population was originally obtained from aubergine, *Solanum melongena* L. (Solanales: Solanaceae), grown in Coimba- tore, South India, and was selected for homozygosity over multiple generations. The population was confirmed as belonging to the Asia I genetic clade by partial sequencing of the mitochondrial cytochrome c oxidase 1 (mtCO1) gene according to established methods ( Dinsdale et al. 2010 ). The insects were held under Invertebrate DEFRA-licence Number PHL 176C/6274 (03/2010).

### Experimental samples

To obtain temporal and spatial gene expression profiles of the reference gene transcripts during postembryonic development in *B. tabaci* , we analysed tissue from six different developmental stages/tissue types: eggs, first instars, fourth instars (red eye puparia), adults, dissected heads plus thorax (with legs and wings removed), and dissected abdomen. The samples taken from the whitefly colony comprised both males and females. Whitefly tissues were dissected under a binocular microscope with typically 1–5 mg of tissue from each stage being collected and frozen immediately in liquid nitrogen and stored at -80°C. For each developmental stage/tissue type, two biological replicates were used (separately) for RNA extraction. To obtain gene expression profiles in response to thermal stress, approximately 200 *B. tabaci* adults (comprised of both males and females) were collected for each sample and incubated for 3.5 hr at different temperatures ranging from 1°C to 15°C with a gradient of 7°C, and from 25°C to 45°C with a gradient of 5°C. Following treatment, the samples were immediately frozen in liquid nitrogen and stored at -80°C. For each temperature treatment, two biological replicates were used (separately) for RNA extraction. To minimise the impact of potential RNA degradation on results, only RNA samples stored for less than two weeks were used in our study.

### Total RNA extraction and cDNA synthesis

Total RNA was extracted from *B. tabaci* tissue using TRIzol Reagent (Invitrogen, www.lifetechnologies.com ) and eluted using 40 µL nuclease-free water (Thermo Scientific, www.thermoscientific.com ). Additional reagents required for extraction (isopropanol, ethanol, chloroform) were obtained from Sigma-Aldrich ( www.sigmaaldrich.com ). RNA was further purified using the RNAque- ous-4PCR kit with subsequent DNaseI treatment on the column (Ambion, Invitrogen) to eliminate potential genomic DNA contamination. RNA concentration and purity in each extract were determined using a BioPhotome- ter (Eppendorf, www.eppendorf.com ). Purity was calculated from the 260/280 nm ratio, and only those samples with values of between 1.8 and 2.1 were used in this study. To confirm the absence of significant RNA degradation, RNA integrity was visualised by denaturing agarose gel electrophoresis and ethidium bromide staining. Reverse transcription was performed using 2 µg total RNA in a total reaction volume of 20 µL with the RETROscript kit (Ambion) utilising an oligo (dT)18 primer. Following an initial treatment at 70°C for 3 min, the RT reaction was carried out at 42°C for 1 hr using 1 µL MMLV-RT Reverse Transcriptase (100 units), and terminated by heating to 92°C for 10 min. After cDNA synthesis, all cDNA samples were diluted 10 times in molecular grade water and stored at -20°C.

### Identification of reference genes and primer design

**Figure S1.**
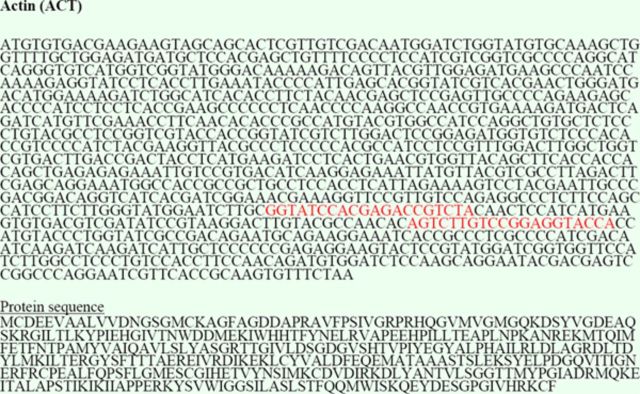

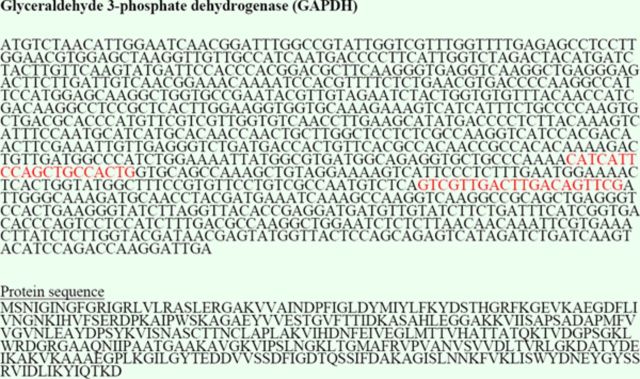

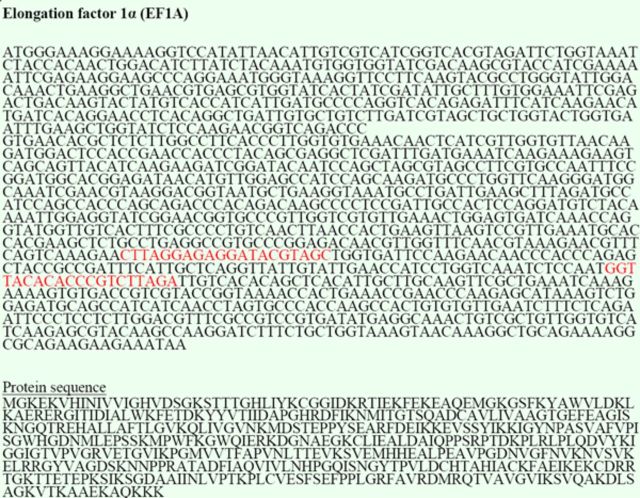

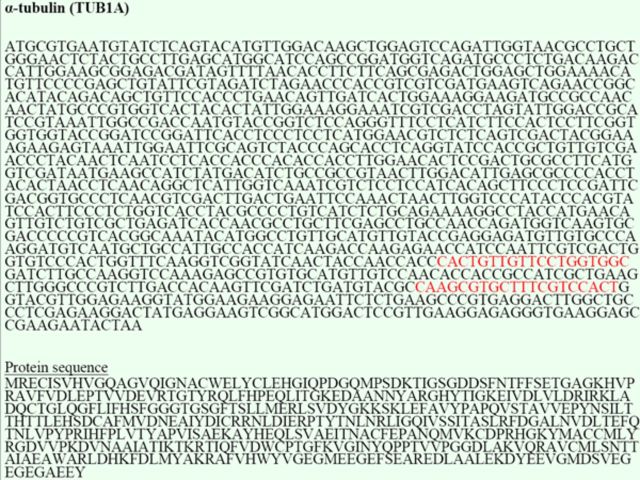

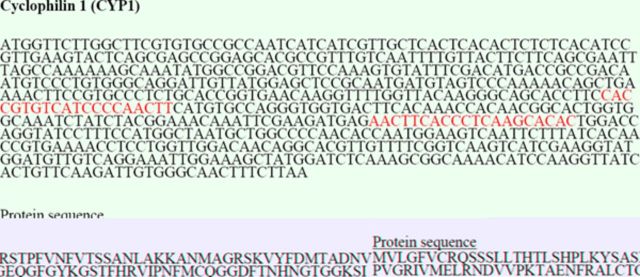

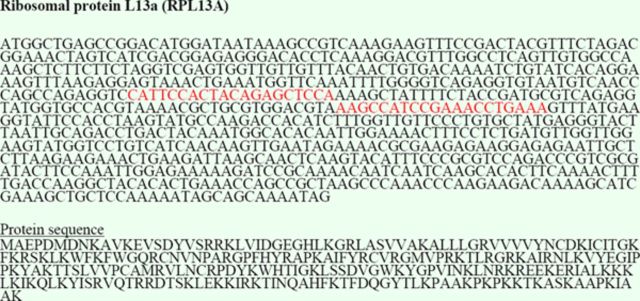

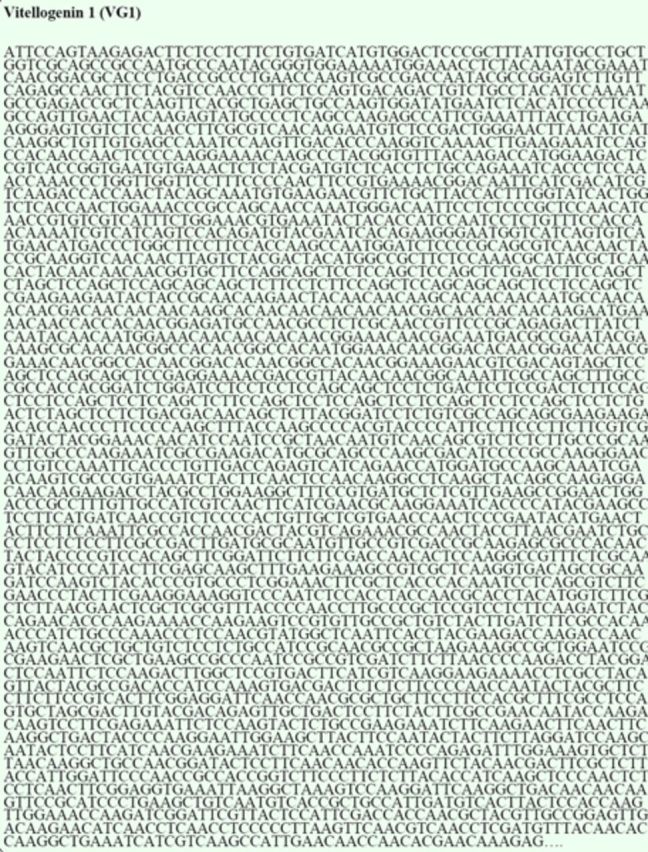

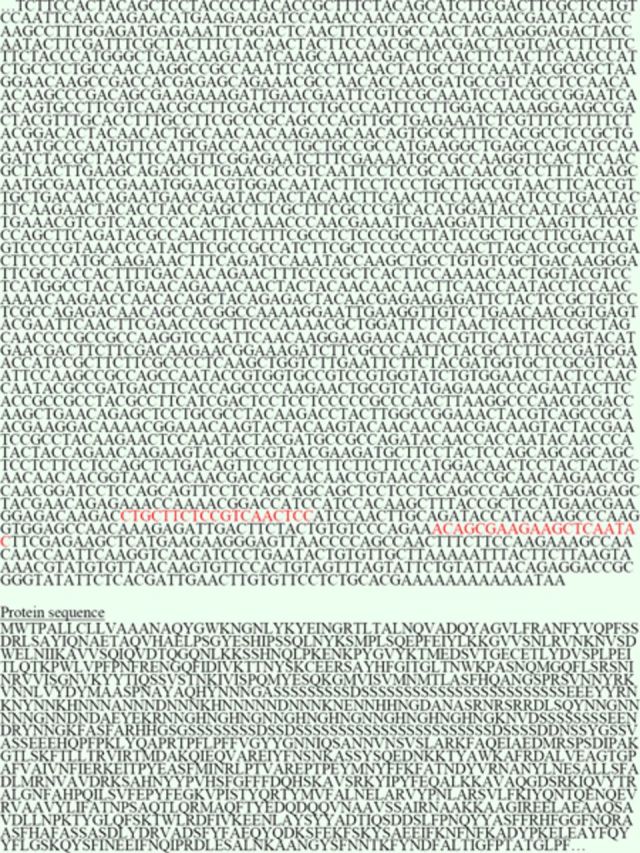

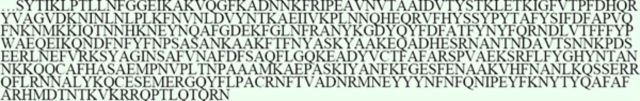
DNA sequences (coding domain sequences and translated protein) of the *B. tabaci* reference and target genes identified in this study. The primer sequences used for RT-qPCR are highlighted underlined in red. High quality figures are available online.

Potential candidate reference genes from *Drosophila melanogaster* (Meigen) (Diptera: Drosophilidae) and other closely related insects were used for nucleotide and protein BLAST searches among the available expressed sequence tag databases of *B. tabaci* ( Leshkowitz et al. 2006 ; Wang et al. 2010 ) and from our own *B. tabaci* Asia I sequence collection ( Seal et al. 2012 ). Sequences for six common reference genes were identified in *B. tabaci* : actin (ACT), cyclophilin 1 (CYP1), elongation factor 1α (EF1A), glyceraldehyde 3-phosphate dehydrogenase (GAPDH), ribosomal protein L13a (RPL13A), and α-tubulin (TUB1A) ( Table 1 , Figure S1 ). In addition, one sequence that showed high homology to vitellogenin (VG) genes in other insects was identified in the above databases and used in this study as a developmental marker to further validate the gene expression profiles determined during development ( Table 1 , Figure S1 ). To prevent co-amplification of homologous target sequences, multiple sequence alignments were performed using ClustalW (EMBL-EBI, www.ebi.ac.uk/clustalw/ ), and Asia I-specific, non-degenerate primers were designed to amplify a single product from each of the reference genes and target gene ( Figure S1 ). Primer sequences and the characteristics of each primer pair used in this study are shown in Table 2 . All primers were designed with the aid of the OligoCalc software ( www.basic.northwestern.edu/biotools/oligocalc.html ) and synthesized and purified (by de- salting) at Invitrogen (UK). PCR amplification conditions were first optimised using standard PCR with AmpliTaq Gold 360 (Applied Biosystems, www.appliedbiosystems.com ) to determine optimal primer and MgCl2 concentrations and thermocycling parameters. Specificity of all primers for their respective target sequence was demonstrated by (1) the amplification of a single PCR product followed by sequencing of the resulting PCR fragment (GeneService UK, www.lifesciences.sourcebioscience.com ), (2) the generation of a single peak in the dissociation (melting curve) analysis following RT-qPCR (below), and (3) an *in silico* specificity screen using BLAST analysis.

**Table 1.**
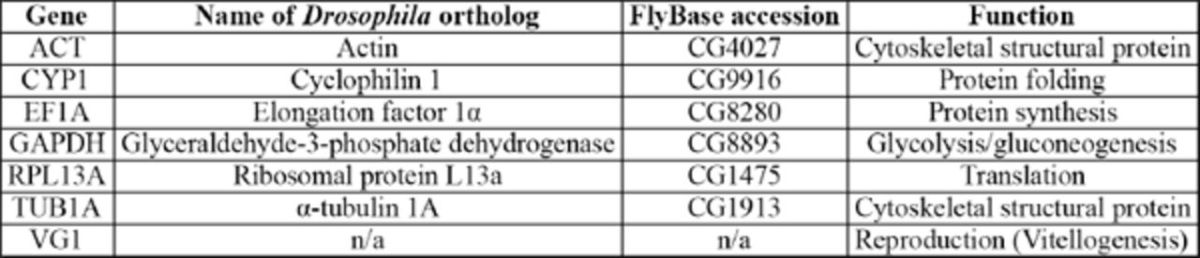
Candidate reference and target genes evaluated in this study.

n/a = not applicable.

**Table 2.**
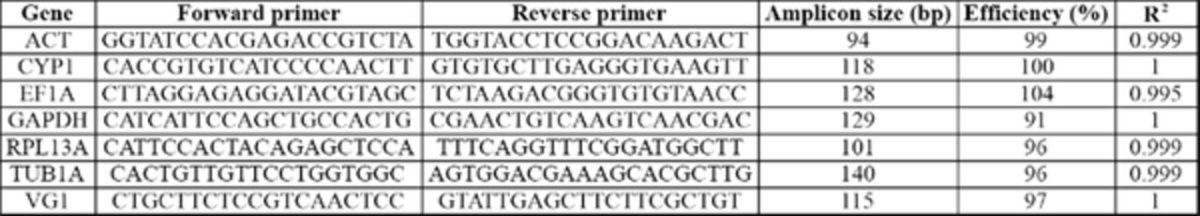
Information on the oligonucleotide primers used in RT-qPCR.

R2 = correlation coefficient.

### Reverse transcription quantitative realtime PCR assays

All RT-qPCR reactions were prepared using an ep *Motion 5070* robotic dispenser (Eppendorf) in twin.tec 96-well PCR plates (Eppendorf). Each 25 µL reaction consisted of 11.25 µL RealMasterMix SYBR ROX 2.5x (5Prime, www.5prime.com ), 1.0 µL forward and reverse primer (final concentration of 200 nm each primer), 2.5 µL diluted cDNA, and 10.25 µL molecular biology grade water. Assays were also performed in parallel in a number of experiments using the QuantiTect SYBR Green PCR Kit (Qiagen, www.qiagen.com ) and gave equally satisfactory results (data not shown). All samples were analysed in the same run and reactions were performed in triplicate on a Mastercycler ep *realplex* cycler (Eppendorf) using the following thermal cycling profile: 95°C (15 min) followed by 40 cycles of 95°C for 15 sec, 60°C for 30 sec, and 68°C for 30 sec. Finally, a dissociation (melting curve) analysis was performed to confirm the amplification of a single expected PCR product and the absence of primer dimer formation, according to the manufacturers’ instructions (Eppendorf). To obtain amplification efficiencies for each primer set, a relative standard curve was generated for each primer pair using 10-fold serial dilutions of cDNA corresponding to 500 ng to 0.5 ng cDNA and analysed using the *realplex* software (Eppendorf). Efficiency of amplification (E) and correlation coefficients (R2) were calculated for each primer pair. PCR efficiencies derived in this study are shown in Table 2 . Several minus RT (negative control) reactions were included with primers specific for ACT, GAPDH, and EF1A to confirm successful removal of all contaminating DNA in all RNA samples as shown by the failure to detect the corresponding amplicon in each minus RT control reaction by RT-qPCR. Furthermore, additional negative control (no template control) reactions (NTC) were included in all assays by substituting water for template DNA to confirm that no contaminating DNA was present in any of the PCR reagents.

### Data analysis

The stability of the candidate reference genes during development and in response to thermal stress was determined using three different software packages: geNorm ( Vandesompele et al. 2002 ) (downloaded from http://medgen.ugent.be/~jvdesomp/genorm/ ), NormFinder ( Andersenet al. 2004 ) ( www.mdl.dk/publicationsnormfinder.htm ), and BestKeeper ( Pfaffl et al. 2004 ) ( www.gene-quantification.com/bestkeeper.html ). GeNorm conducts pairwise comparisons and ranks the reference genes based on the similarity of their expression profiles such that those genes with the most similar expression profiles will be ranked the highest independent of experimental conditions but is susceptible to the inclusion of coregulated genes. NormFinder applies a mathematical model to estimate the variation of the candidate reference genes (intra- and inter-group expression variations) and is less susceptible to coregulated genes because it will identify the most stable genes as those with the least overall variation in expression. BestKeeper ranks the reference genes according to expression stability based on calculations of the standard deviations and coefficient of variance of expression levels and identifies the best genes as those with least variation. For both geNorm and NormFinder, it was necessary to transform the raw averaged quantification (threshold) cycle (Cq; terminology according to established guidelines ( Bustin et al. 2009 )) values of triplicate reactions to quantities using the comparative Ct method and specific PCR efficiencies in order to provide an input file format suitable for analysis using these two Microsoft Excel ( www.microsoft.com ) applications according to the described methods ( Vandesompele et al. 2002 ; Andersen et al. 2004 ). For the BestKeeper application, the raw averaged Cq values were used (without transformation) to calculate the gene stability values according to the instructions contained with the program. To determine the relative (normalised) expression level of the vitellogenin (VG1) gene, we used both the ∆∆Ct method ( Pfaffl 2001 ) and the geNorm program ( Vandesompele et al. 2002 ), which each had different input requirements. The ∆∆Ct method requires the raw Cq values for input, uses a single reference gene for normalisation, and displays the transcript abundance of the target gene relative to a designated 1-fold control sample (usually the sample with the lowest expression). GeNorm, on the other hand, requires transformed Cq values for input (discussed above), uses the geometric mean of selected reference gene quantities to calculate a normalisation factor, and shows the normalised expression levels relative to a designated 1-fold control sample. Statistical tests of significance for gene expression changes using raw Cq values was determined by ANOVA in SPSS software (IBM, www.ibm.com ) with a threshold of *P* < 0.01.

## Results

### Reverse transcription quantitative realtime PCR assay development

**Figure S2.**
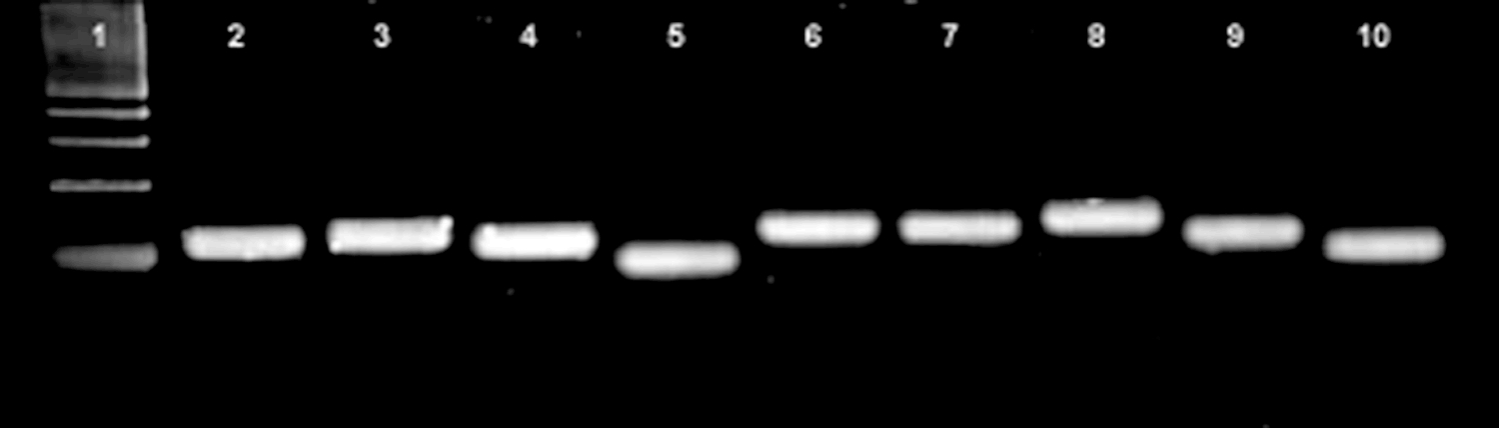
PCR amplification products generated using gene- specific primers for reference and target genes. PCR products were resolved by gel electrophoresis in a 3% agarose gel and stained using ethidium bromide. Lanes: (1) 100 bp marker (New England Biolabs) (2) Not specified (not applicable) (3) Not specified (not applicable) (4) VG1 (5) ACT (6) GAPDH (7) EF1A (8) TUB1A (9) CYP1 (10) RPL13A. High quality figures are available online.

From our *B. tabaci* Asia I sequence collection ( Seal et al. 2012 ) and publicly available expressed sequence tag collections ( Leshkowitz et al. 2006 ; Wang et al. 2010 ), we identified and characterised six candidate reference genes in the genome of *B. tabaci* : actin (ACT), cyclophilin 1 (CYP1), elongation factor 1α (EF1A), glyceraldehyde 3-phosphate dehydrogenase (GAPDH), ribosomal protein L13a (RPL13A), and α-tubulin (TUB1A) (Table 1, Figure S1 ). We also identified a sequence in our database that showed a high degree of similarity with vitellogenin (VG) genes from closely-related insects and named it VG1 ( Table 1 and Figure S1 ). From the above sequence collections, we designed and tested gene-specific primers to target each reference gene and the target gene ( Table 2 , Figure S1 ). Initial screening of the six potential reference genes and the target gene by conventional RT-PCR showed that all genes were expressed in adult whiteflies as evidenced by the generation of single amplicons of the expected sizes ( Figure S2 ). Gene- specific amplification of all genes was confirmed by sequencing of the PCR product, and all genes displayed 100% identity with the sequences upon which the primer design was based ( Figure S1 ). The efficiencies of the RT- qPCR amplifications for all seven genes were uniformly high, ranging from 91% to 104%, making all primer pairs suitable for quantitative analysis ( Table 2 , Table S1 ). Under RT- qPCR conditions, all PCR reactions generated a single peak in the realtime dissociation (melting curve) analysis, and the absence of primer dimer formation confirmed the specificity of the gene-specific primers (data not shown).

**Table S1.**
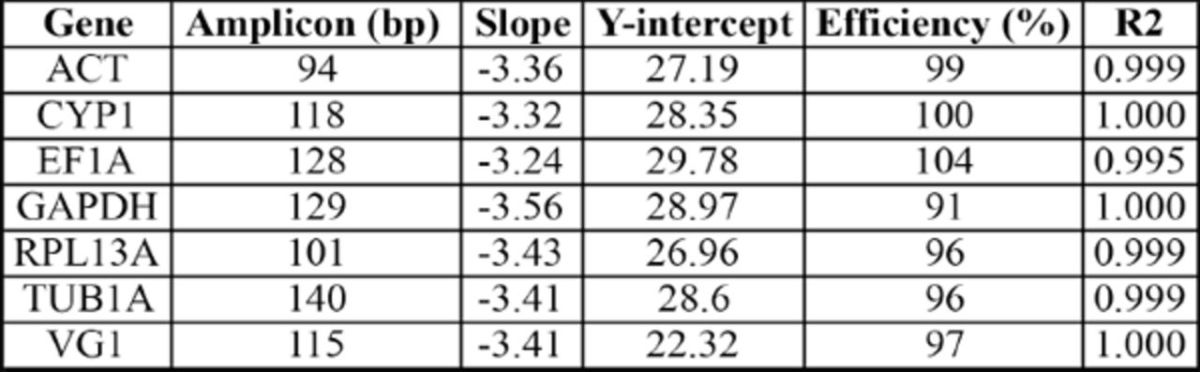
PCR efficiencies for each assay as calculated from dilution (calibration) curves.

R2 = correlation coefficient

### Validation of reference genes during B. tabaci development and in different tissues

**Figure 1.**
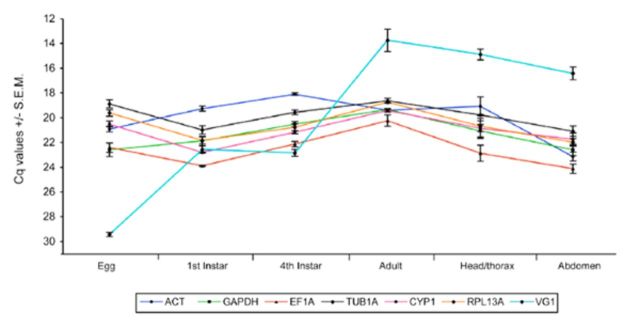
Reference and vitellogenin gene expression during development in *Bemisia tabaci.* For clarity, the y-axis has been reversed to show maximum levels of transcript abundance at different developmental stages. Note also that sample points are joined for clarity in order to show the general trend of gene expression stability for all genes across developmental stages/tissue types and in no way assumes a continuous data set. High quality figures are available online.

**Transcription profiling of reference and target genes** . The RNA transcript levels of the six reference genes and the target gene were monitored over different representative developmental stages and tissue types of *B. tabaci* : eggs, first instars, fourth instars (red eye puparia), adults, adult heads plus thorax, and abdomen. Raw Cq values were used to construct the transcription profiles for all these genes ( Table S2 , Figure 1 ). Overall, Cq values ranged from 18.09 (ACT) to 24.12 (EF1A) for the six reference genes, and the lowest Cq variation was shown for reference gene TUB1A, with ACT showing the most variation ( Table 3 ). All reference genes were uniformly highly expressed in *B. tabaci* with the exception of EF1A, which was expressed at a slightly lower level with a mean Cq value above 22 cycles.

**Table S2.**
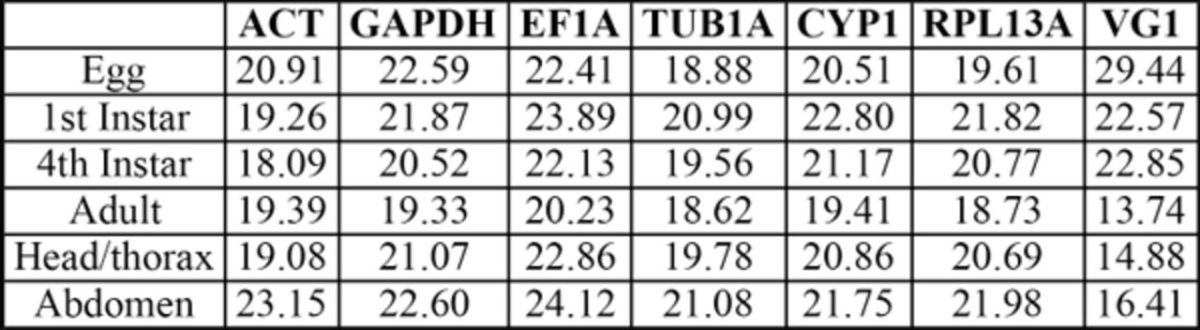
Raw data of the RT-qPCR experiments showing calculated average quantification cycle (Cq) values for reference and target genes obtained during *Bemisia tabaci* development

The VG1 target gene showed a large variation in Cq values ranging from 13.74 to 29.44, with lowest expression during the egg stage (29.44) and the highest expression during the adult stage (13.74; Figure 1 ). The transcription profile of the VG1 gene clearly shows developmental regulation of VG1 transcription and is consistent with a primary functional role in reproduction (vitellogene- sis), as reported in other insects ( Tufail and Takeda 2008 ). This pattern of expression for VG1 is in agreement with data reported for two putative VG-like genes from a recent microarray study in *B. tabaci* ( Wang et al. 2010 ). Therefore, as a developmental marker gene for our RT-qPCR experiments, we believe our data accurately reflects the transcriptional activity for VG1 during *B. tabaci* development and in different tissues, and thus strengthens the validation approach for the reference genes adopted in this study.

**Table 3.**
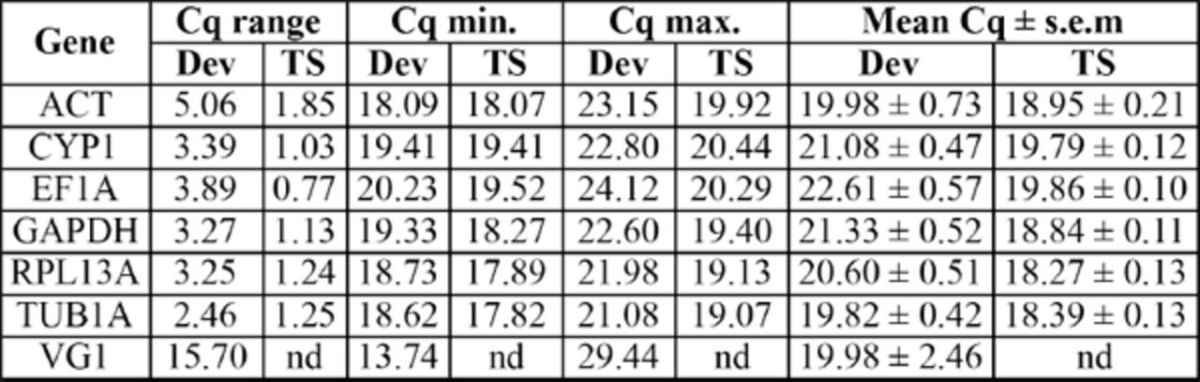
Quantification cycle (Cq) values of candidate reference genes and target gene during development and in response to thermal stress in *Bemisia tabaci* .

Dev = development; TS = thermal stress; nd = not determined.

**Table 4.**
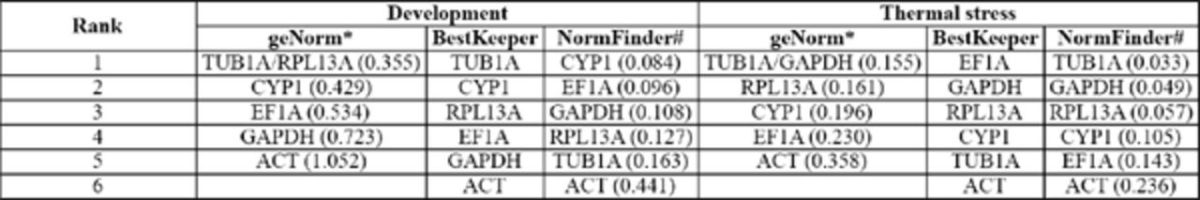
Ranking of the reference genes in order of their expression stability derived from the analyses by geNorm, BestKeeper and NormFinder.

The genes are ranked by decreasing expression stability from top to bottom.

*Average expression stability M values calculated by geNorm shown in parenthesis.

#Stability values calculated by NormFinder shown in parenthesis.

**Evaluation of expression stability** . The expression stability of the six candidate reference genes over representative stages of the life cycle of *B. tabaci* was evaluated using the three software programs BestKeeper, geNorm, and NormFinder. The geNorm approach ranked the reference genes according to the average expression stability M (AESM) values determined, from the most stable (lowest M value) to the least stable (highest M value) gene ( Table 4 ). GeNorm identified RPL13A and TUB1A as the most stable endogenous control gene, with a combined AESM value of 0.355, followed by CYP1, which increased the AESM value to 0.429 for the combination of the best three genes. However, all genes reached a rather high expression stability, with AESM values below the accepted limit of M = 1.5 ( Vandesompele et al. 2002 ). NormFinder ranked CYP1 and EF1A as the two most stably-expressed genes according to the lowest observed stability values, followed by GAPDH ( Table 4 ). The analyses conducted with BestKeeper ranked the reference genes based on the standard deviation of the Cq values and identified the same three best genes as with geNorm, with the most stable gene being TUB1A, followed by CYP1 and RPL13A as second and third gene ( Table 4 , Table S3 ). Taken together, the analyses using all three software programs identified TUB1A, RPL13A, and CYP1 as the three most stably expressed genes during *B. tabaci* development. ACT was found to be the least stable gene in all stages and tissues by all three programs.

**Table S3.**
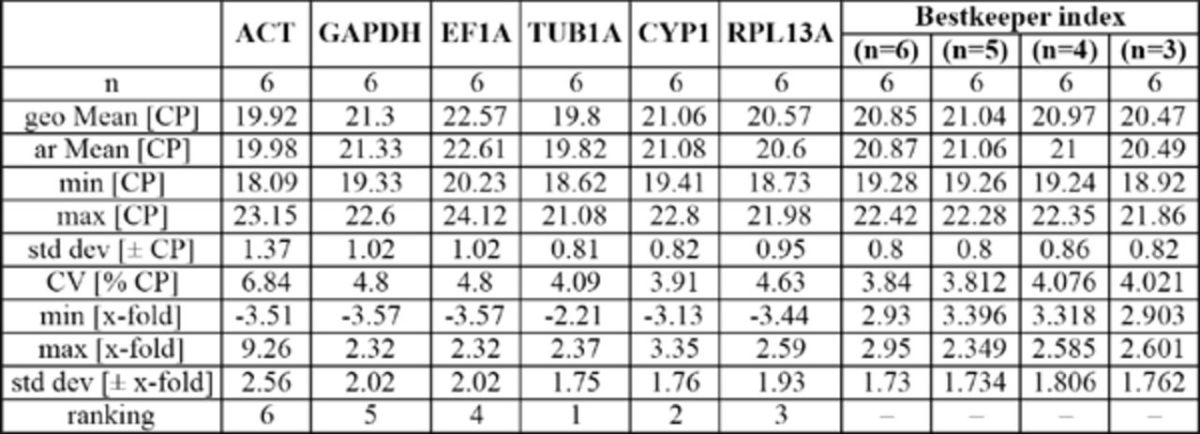
Ranking of the reference genes during *Bemisia tabaci* development using BestKeeper software.

**Figure 2.**
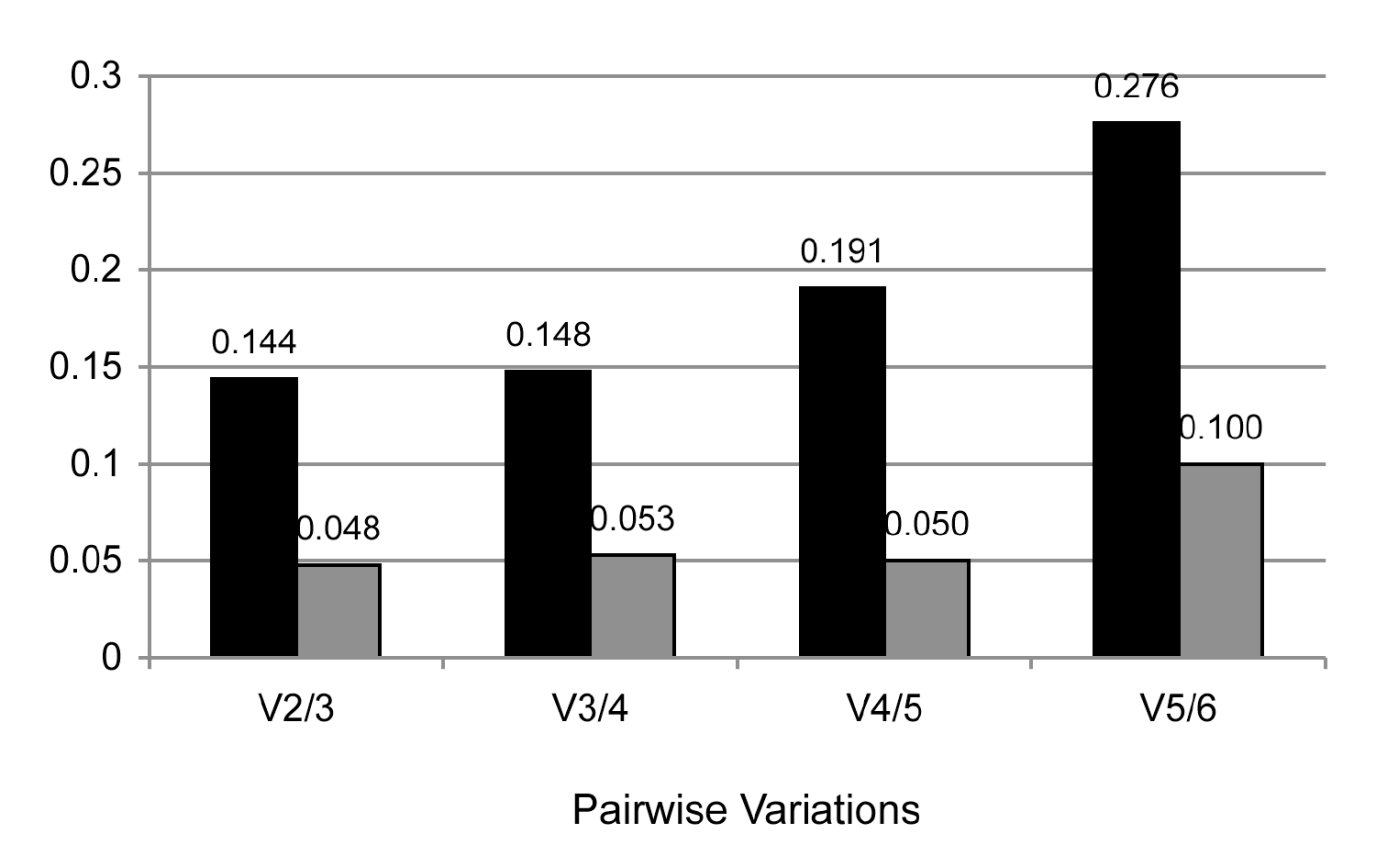
Pairwise variation analysis to determine the optimal number of reference genes required for accurate normalisation. Pairwise variation analysis between the normalisation factors NFn and NFn+1 was performed to determine the optimal number of reference genes required for accurate normalisation during development (black bars) and in response to thermal stress (grey bars) in *Bemisia tabaci* using geNorm software. High quality figures are available online.

The minimum number of genes necessary for accurate normalisation was determined from the calculation of the pairwise variation (Vn/n+1) using geNorm ( Figure 2 ). A decreasing variation in this ratio as a result of inclusion or removal of a candidate gene from the analysis corresponds to increasing expression stability. The lowest V value was found to be 0.144 for V2/3 and is below the widely- accepted default cut-off of 0.15 (Vandesompele et al. 2002) indicating that two reference genes, TUB1A and RPL13A, would be sufficient to accurately normalise the data obtained during development. However, since NormFinder and BestKeeper found CYP1 overall more stable than RPL13A ( Table 4 ) and CYP1 was ranked third by geNorm, CYP1 should be included with TUB1A and RPL13A in the set of the three best reference genes. The inclusion of CYP1 gave a V value of 0.148 for V3/4, below the 0.15 cut-off indicating a suitably stable expression level for all three genes across developmental samples ( Figure 2 ).

### Validation of reference genes in adult B. tabaci exposed to thermal stress

**Figure 3.**
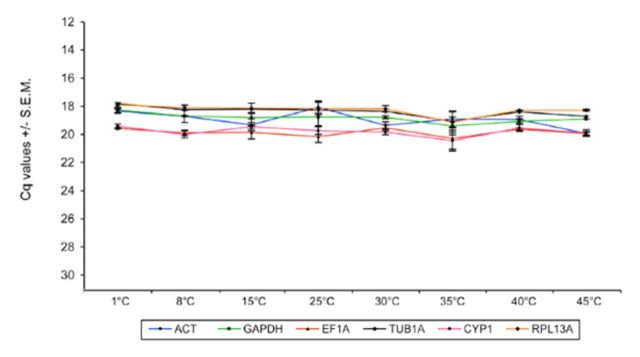
Reference gene expression in *Bemisia tabaci* adults responding to thermal stress. Note that the y-axis has been reversed as in Figure 1 . Note also that the scale of the x and y-axis used (minimum and maximum values set) are the same as for Figure 1 to highlight the relatively high expression stability of the reference genes under thermal stress treatment compared to the expression levels of the respective genes during development. High quality figures are available online.

**Transcription profiling of reference genes** . Transcription profiles were generated for the six reference genes in adults of *B. tabaci* exposed to different temperatures ( Figure 3 , Table S4 ). Collectively, the variation in transcript levels of the endogenous controls in thermally stressed adults were significantly lower than those during development( Table 3 , Figure 3 ). The lowest Cq variation among the reference genes across all samples was observed for EF1A. ACT showed the greatest overall variation in transcript levels.

**Table S4.**
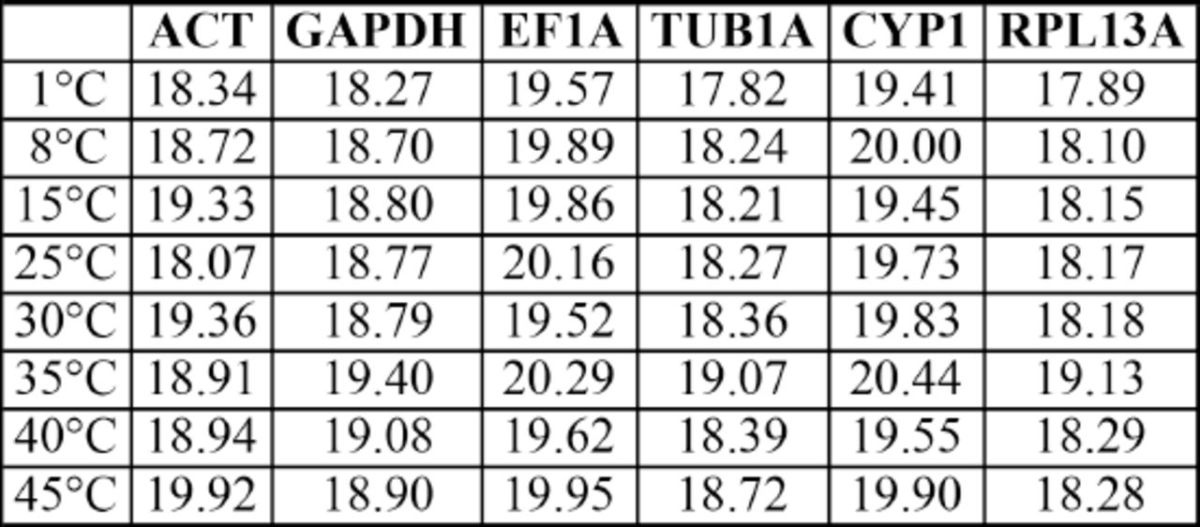
Raw data of the RT-qPCR experiments showing calculated average quantification cycle (Cq) values for reference genes obtained under thermal stress treatment.

**Evaluation of expression stability** . The three software programs all showed the stability values calculated for the genes during thermal stress to be significantly higher than for those obtained during development ( Table 4 ). The geNorm analysis showed TUB1A and GAPDH to be the most stable reference genes and RPL13A to be ranked third ( Table 4 ). All reference genes showed AESM values below the default limit of M = 1.5 ( Vandesompele et al. 2002 ), which is indicative of a high expression stability during thermal stress. NormFinder indicated the same three best genes as with geNorm, with TUB1A being most stable, followed by GAPDH and RPL13A as second and third best ( Table 4 ). According to BestKeeper, the most stable reference gene was EF1A, followed closely by GAPDH and RPL13A ( Table 4 , Table S5 ). By the combination of all three programs, TUB1A, GAPDH, and RPL13A emerge as the three most stably-expressed genes in adult *B. tabaci* exposed to thermal stress. ACT was found to be the least stable gene by all three programs.

**Table S5.**
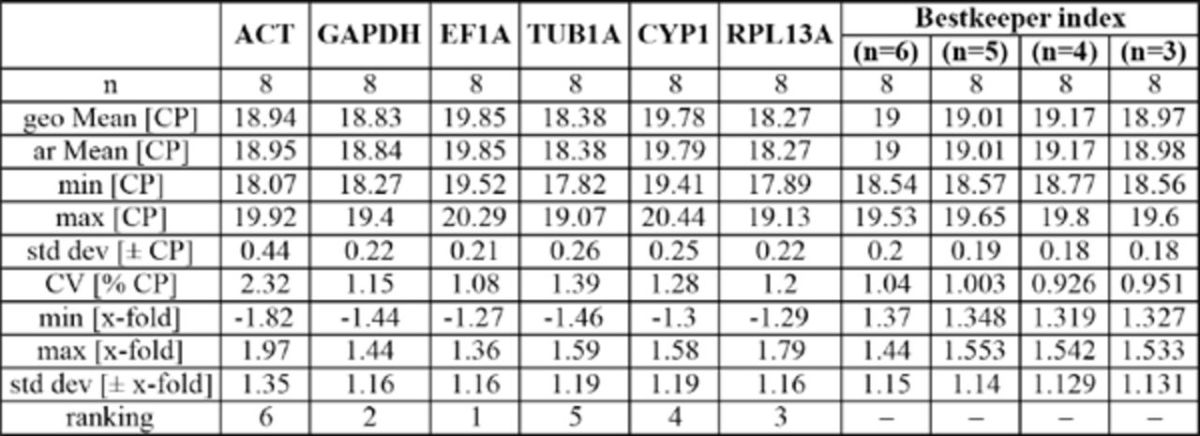
Ranking of the reference genes in *Bemisia tabaci* adults exposed to thermal stress using BestKeeper software.

The minimum number of genes necessary for accurate normalisation of RT-qPCR data during thermal stress was calculated using geNorm ( Figure 2 ). The lowest V value was found to be 0.048 for V2/3, which and is well below the default cut-off of 0.15 (Vandesompele et al. 2002). This implies that two reference genes, TUB1A and GAPDH, would be sufficient to accurately normalise the data obtained during thermal stress. Because TUB1A and GAPDH are ranked the overall best two genes by the combination of all three software programs ( Table 4 ), it is suggested to employ these genes for normalisation of data obtained during thermal stress. As RPL13A was ranked third overall, this gene could be included as an optional third reference gene. Its inclusion would have only a minimal effect on the calculated V value (0.053 for V3/4; Figure 2 ).

### Effect of reference gene selection on relative expression profile of VG1

**Figure 4.**
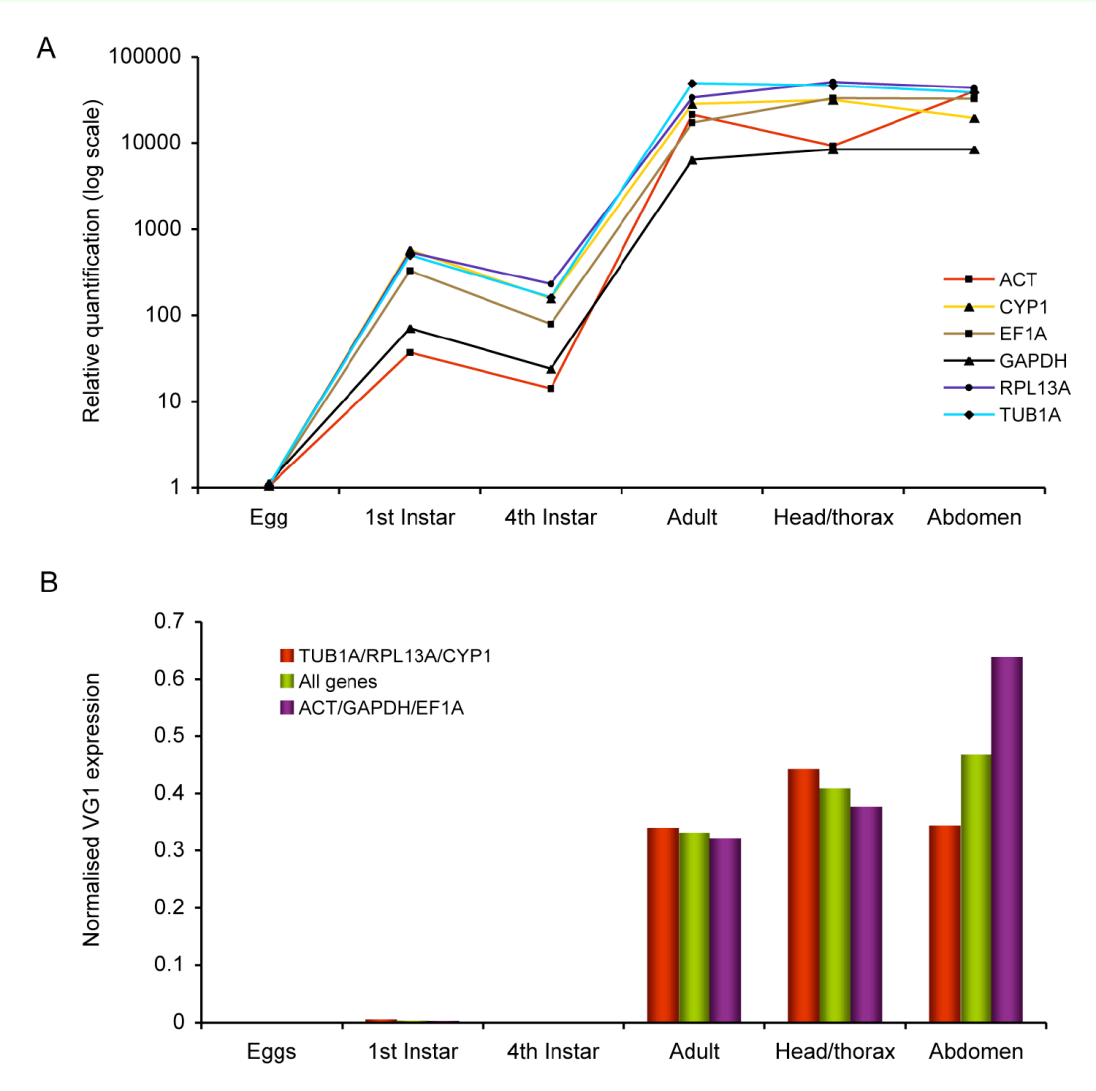
VG1 expression level during *Bemisia tabaci* development and in different tissues normalised under different sets of reference genes. (A) Relative quantification (log scale) of the VG1 gene normalised separately against each of the six reference genes using the ∆∆Ct method ( Pfaffl 2001 ) with the egg stage of development set as 1-fold expression. (B) VG1 expression levels normalised with three different sets of reference genes: the best three identified from this study (TUB1A/RPL13A/CYP1 (red bars)), all six reference genes (purple bars) and the worst three identified (ACT/GAPDH/EF1 (green bars)). Relative expression levels were calculated using geNorm ( Vandesompele et al. 2002 ) with the adult stage of development set as 1-fold expression. Note that sample points are joined for clarity in order to show the general trend of gene expression stability for all genes across developmental stages/tissue types and in no way assumes a continuous data set. High quality figures are available online.

Because we observed significant variation in the transcript levels of the candidate reference genes across developmental stages and tissue types of *B. tabaci,* we decided to examine the effects of reference gene selection on the calculated relative expression level of the VG1 gene during *B. tabaci* development. First, we normalised the VG1 expression data using each of the six genes separately to obtain relative quantification profiles. The relative quantification for VG1 expression showed significant variation in all stages and tissue types (oneway ANOVA, *P* < 0.01), mostly due to normalization with ACT and GAPDH, which presented the largest variation ( Figure 4A ). These two reference genes were found to be the overall least stable genes during development according to the analyses with geNorm, NormFinder, and BestKeeper ( Table 4 ). In contrast, normalisation with TUB1A and RPL13A presented the smallest variation and resulted in highly similar expression profiles. Next, we normalised VG1 expression with three different sets of reference genes: (A) with the three most stable genes identified from this study (TUB1A, RPL13A, CYP1), (B) with all six genes used in this study, and (C) with the three least stable genes identified in this study (ACT, GAPDH, EF1). Each set of reference genes showed the expected upregulation of the VG1 gene at the adult stage and in adult tissues as compared to the egg and instar stages, as well as a significant upregulation from adults to adult head/thorax ( Figure 4B ). However, whereas VG1 expression was seen to be significantly down-regulated from the head/thorax sample to the abdomen sample when using the three best (most stable) reference genes, normalisation using all six endogenous controls showed VG1 expression to be up-regulated. An even greater difference in VG1 expression was observed for normalisation with the three worst (least stable) reference genes, which presented an even larger degree of upregulation between the head/thorax and abdomen samples. Normalisation with ACT, GAPDH, and EF1A clearly overestimated the expression level for VG1 by approximately 2.5-fold compared to normalisation with TUB1A, RPL13A, and CYP1. These results demonstrate that reference gene selection can have a major influence on the calculated expression levels of target genes under specific experimental conditions.

## Discussion

It is now widely accepted that the generation of accurate and reliable gene expression measurements by RT-qPCR is best achieved through the employment of candidate normalisation genes whose expression has been shown to be stable under the experimental conditions to be examined ( Vandesompele et al. 2002 ; De Jonge et al. 2007 ). Surprisingly, validation studies of reference genes in insects are still rare and currently limited to *Apis mellifera* ( Lourenço et al. 2008 ; Scharlaken et al. 2008 ), *Schistocerca gregaria* ( Van Hiel et al. 2009 ), *Folsomia candida* and *Orchesella cincta* ( De Boer et al. 2009 ), *Tribolium castaneum* ( Lord et al. 2010 ; Toutges et al. 2010 ), *Bombyx mori* , *Plutella xylostella* , *Chilo sup-pressalis,* and *Spodoptera exigua* ( Xie et al. 2012 ). Herein, we addressed the current requirement for standardisation of RT-qPCR data normalisation in the whitefly *B. tabaci* by evaluating the suitability of a set of candidate reference genes for RT-qPCR analyses during development and in response to thermal stress. In previous RT-qPCR studies in this species, data normalisation has been based on the use of a single, non-validated gene, most of which have been chosen from the set of classical ‘housekeeping genes’ commonly-used in other organisms, such as actin ( Sinisterra et al. 2005 ; Alon et al. 2008 ; Mahadav et al. 2008 ; Yu and Wan 2009 ), 18S rRNA ( Mason et al. 2008 ), and tubulin ( Mahadav et al. 2009 ). Despite the fact that the transcript levels of many commonly-used reference genes can vary significantly under particular experimental conditions ( Radonić et al. 2004 ; De Jonge et al *.* 2007 ), and that the use of single endogenous controls can lead to highly erroneous estimates of expression levels ( Vandesompele et al. 2002 ; De Jonge et al. 2007 ), there has been a continued reliance on a single, non- validated normalisation gene that threatens to undermine the validity of previous RT-qPCR analyses in *B. tabaci.* In a systematic approach to select the most suitable reference genes for RT-qPCR analyses in *B. tabaci* , we identified putative orthologs of six commonly-used endogenous control genes in the *B. tabaci* genome and analysed their stability at key transitional stages during the life cycle, in different tissues, and in adults exposed to thermal stress at different temperatures using the three statistical programs geNorm, NormFinder, and BestKeeper ( Vandesompele et al. 2002 ; Anderson et al *.* 2004; Pfaffl et al. 2004 ). These three software programs differ in the underlying mathematical models they adopt, so we applied all three programs to our data in order to provide a robust screening approach to selection of reference genes based on consensus.

The data from our study showed that the expression level of reference genes fluctuated much more between developmental samples than those from thermal stress, suggesting that there is a greater degree of modulation in the transcriptional activity for some, if not all, of the reference genes during transitions from one developmental stage to another (and between tissues) compared to thermal stress. This is not unexpected, because the change from one morphological form to another involves widespread physiological changes that are accompanied by global genetic and epige- netic changes in gene regulatory pathways. During development, TUB1A, RPL13A, and CYP1 were found to be the most stable genes, although peculiarly RPL13A and TUB1A were scored as fourth and fifth most stable, respectively, by NormFinder. This software program adopts a different statistical algorithm for calculating stability, and the fact that the gene set showed rather high expression stability under the NormFinder analysis (except for ACT) may have prevented any consensus being reached between the software approaches. Interestingly, GAPDH performed poorly in the analyses, being ranked second to last overall, whereas ACT emerged as the least stable gene by all programs. These results appear to be in contrast to validation studies in locust that revealed TUB1A as being one of the worst genes during locust development, but found ACT and GAPDH to be most suitable ( Van Hiel et al. 2009 ). ACT was scored as a similarly stable gene in a validation study of reference genes during development in *Apis mellifera* ( Lourenço et al. 2008 ).

The analyses for adults exposed to thermal stress revealed that TUB1A and RPL13A were again among the three most stable reference genes, along with GAPDH. ACT was once again ranked the least stable gene by all three programs. According to both geNorm and NormFinder, TUB1A was ranked first, whereas it came fifth using BestKeeper. The discordance in ranking is likely to be attributable to the very high expression stability of the reference genes during the temperature treatments, thus preventing the same consensus from being attained by all three software programs, as noted previously for developmental samples. This is supported by the closeness in stability measurements (standard deviations and coefficients of variance) observed between all genes using the BestKeeper approach. The high ranking of GAPDH in this case was surprising given that it was one of the worst genes identified during development, demonstrating that not every gene shown to be suitable in one set of experimental conditions can be considered ideal automatically under a different experimental regime. The results obtained for thermal stress are in accordance with reference gene analyses in the springtail, *O. cincta* , which found TUB1A as among the most stable in response to cadmium treatment, starvation, and in a combined analysis following various chemical and physical stresses ( De Boer et al. 2009 ). Similarly, GAPDH was ranked as one of the best reference genes in the honey bee following a bacterial challenge ( Scharlaken et al. 2008 ), whereas RPL13A was identified as an ideal reference gene in *T. castaneum* , both following a fungal challenge ( Lord et al. 2010 ) and across life cycle stages and tissue types ( Toutges et al. 2010 ). In the study of Lord et al. (2010) , ACT was scored as unsuitable because it was among the least stable genes. In complete contrast to our findings, ACT was found to be the most suitable gene in the honey bee ( Scharlaken et al. 2008 ), whereas TUB1A and RPL13A were revealed as the least suitable in the same study.

We illustrated the effects of reference gene choice for obtaining an accurate transcription profile for a developmentally-regulated target gene, vitellogenin1 (VG1). We chose to focus on development rather than thermal stress because we observed greater fluctuations in reference gene mRNA levels during development than thermal stress, thus allowing us to highlight more clearly the consequences of incorrect normalisation on calculated expression. This gene served as an ideal developmental marker to validate the experimental approach for measuring transcript abundance used in our study because the pattern of expression observed for VG1 during development was in accordance with previous reports describing insect VG gene expression ( Tufail and Takeda 2008 ; Wang et al. 2010 ). Although the overall transcription profile for VG1 was similar for whichever normalisation genes were used, selection of different subsets of reference genes gave drastically different quantitative measurements of target gene expression, demonstrating the importance of adopting the correct normalisation strategy for accurate gene expression analysis.

In conclusion, our study provides the first detailed survey on the identification and evaluation of candidate reference genes for accurate and reliable gene expression studies in *B. tabaci* . We propose that TUB1A, RPL13A, and CYP1 should be used in RT- qPCR studies of *B. tabaci* development, whereas TUB1A, GAPDH, and RPL13A are suitable for studies on thermal stress. The validation approach detailed here represents a valuable starting point for assisting the selection of suitable reference genes for future *B. tabaci* gene expression analyses under any experimental conditions. The range of genes optimised for evaluation here can be broadened to include additional genes should it be necessary, a task that will be easier to accomplish once a fully sequenced genome is available for this organism.

## Abbreviations

ACT: actin
AESM: average expression stability M
CYP1: cyclophilin 1
Cq: quantification cycle
EF1A: elongation factor 1α
GAPDH: glyceraldehyde 3-phosphate dehydrogenase
RPL13A: ribosomal protein L13a
TUB1A: α-tubulin
VG: vitellogenin
